# Comparison of HRV indices obtained from ECG and SCG signals from CEBS database

**DOI:** 10.1186/s12938-019-0687-5

**Published:** 2019-06-01

**Authors:** Szymon Siecinski, Ewaryst J. Tkacz, Pawel S. Kostka

**Affiliations:** 1 Department of Biosensors and Biomedical Signal Processing, Faculty of Biomedical Engineering, Silesian University of Technology, 40 Roosevelt’s Street, 41-800 Zabrze, Poland; 2 Katowice School of Technology, 43 Rolna Street, 40-055 Katowice, Poland

**Keywords:** Seismocardiography, Heart rate variability, HRV analysis

## Abstract

**Background:**

Heart rate variability (HRV) has become a useful tool of assessing the function of the heart and of the autonomic nervous system. Over the recent years, there has been interest in heart rate monitoring without electrodes. Seismocardiography (SCG) is a non-invasive technique of recording and analyzing vibrations generated by the heart using an accelerometer. In this study, we compare HRV indices obtained from SCG and ECG on signals from combined measurement of ECG, breathing and seismocardiogram (CEBS) database and determine the influence of heart beat detector on SCG signals.

**Methods:**

We considered two heart beat detectors on SCG signals: reference detector using R waves from ECG signal to detect heart beats in SCG and a heart beat detector using only SCG signal. We performed HRV analysis and calculated time and frequency features.

**Results:**

Beat detection performance of tested algorithm on all SCG signals is quite good on 85,954 beats ($\text {Se}=0.930$, $\text {PPV}=0.934$) despite lower performance on noisy signals. Correlation between HRV indices was calculated as coefficient of determination ($R^2$) to determine goodness of fit to linear model. The highest $R^2$ values were obtained for mean interbeat interval ($R^2 = 1.000$ for reference algorithm, $R^2 = 0.9249$ in the worst case), ${{\text{PSD}}}_{{\text{LF}}}$ and ${{\text{PSD}}}_{{\text{HF}}}$ ($R^2 = 1.000$ for the best case, $R^2 = 0.9846$ for the worst case) and the lowest were obtained for ${{\text{PSD}}}_{{\text{VLF}}}$ ($R^2 = 0.0009$ in the worst case). Using robust model improved achieved correlation between HRV indices obtained from ECG and SCG signals except the $R^2$ values of pNN50 values in signals p001–p020 and for all analyzed signals.

**Conclusions:**

Calculated HRV indices derived from ECG and SCG are similar using two analyzed beat detectors, except SDNN, RMSSD, NN50, pNN50, and ${{\text{PSD}}}_{{\text{VLF}}}$. Relationship of HRV indices derived from ECG and SCG was influenced by used beat detection method on SCG signal.

## Introduction

Heart rate variability (HRV) is the physiological phenomenon of variation of time between heartbeats [1], which is caused by the activity of autonomic nervous system [2]. HRV has been frequently used in the analysis of physiological signals in different clinical and functional conditions [3, 4]. Low HRV is a risk factor for myocardial infarction, angina pectoris, and sudden cardiac death [5–8]. Other applications of HRV analysis include atrial fibrillation [9], brain stroke [10–14], sleep bruxism [15] diagnosis, and assessment the progress of rehabilitation of patients after ischemic brain stroke [16].

HRV is traditionally obtained from electrocardiogram (ECG) [17]. Over the recent years, there has been interest into non-invasive heart rate monitoring without using electrodes [18]. Seismocardiography (SCG) is a technique of recording and analyzing cardiac activity by measuring precordial acceleration. Recordings are taken using accelerometer on subjects in supine position [19]. In the past, SCG was mainly a tool for physiologists, due to the need of complex recording devices [20].

Technological improvements and miniaturization of accelerometers and the availability of low cost computational power have provided the reasons for reconsidering seismocardiography in clinical practice [21, 22]. Various applications have been proposed for SCG, including HRV analysis, detecting heart arrhythmia, and myocardial ischemia [22–24].

The feasibility of HRV analysis using SCG signals has been described earlier in papers [17, 18, 25–28]. Ramos-Castro et al. [18] and Landreani et al. [26, 27] showed that SCG signal acquired by smartphones can be used to perform HRV analysis. Laurin et al. [17] proved the validity of HRV indices obtained from SCG signal and Tadi et al. [25] study showed high correlation between HRV indices obtained from ECG and SCG.

The purpose of this study is to compare HRV indices obtained from SCG and ECG on signals from CEBS combined measurement of ECG, breathing, and seismocardiogram database and to determine the influence of heart beat detector on SCG signals. CEBS database is a multi-channel signal database available at PhysioNet.org [29–31]. A preliminary version of this work was presented in paper [28].

## Materials and methods

### Data set

CEBS database contains 60 multi-channel signals acquired on 20 healthy volunteers. Each recording consists of four channels with a sampling frequency of 5 kHz: ECG (lead I and II), respiratory signal and SCG. Electrocardiogram (ECG) and respiratory signal were registered using Biopac MP36 data acquisition system. ECG (channel 1) was recorded with a bandwidth between 0.05 and 150 Hz and channel 4 (SCG) was recorded using the tri-axial accelerometer LIS334ALH by ST Microelectronics and 0.5–100 Hz bandwidth [29–31].

Subjects were asked to be awake and stay still in supine position on a bed during the measurement. After attaching the sensors, the basal state (before playing the music) was acquired for 5 min (recordings b001–b020). Then, the subjects started listening to music for 50 minutes (recordings m001–m020). Finally, the subjects were monitored for 5 min after the music ended (recordings p001–p020) [29, 31].

### ECG signal processing

Several heart beat detectors have been proposed for ECG, which detect QRS complexes [32, 33]. In this study, we applied Pan–Tompkins algorithm [33] implemented by Wedekind [34] to detect R waves in ECG lead I. Pan–Tompkins algorithm consists of the following steps: band-pass filtering (to reduce noise, baseline wandering, muscle noise, etc.), differentiation, squaring of samples, moving average filtering, and correlation analysis [32, 33]. After preprocessing, amplitude thresholding is applied to identify R waves in the ECG signal. The interbeat intervals are calculated as differences between time of occurrence of successive R waves as in the following equation:

1 $$\begin{aligned} t_{\text{RR}, i} = t_n - t_{n-1}, \end{aligned}$$

where $t_{{\text{RR}},i}$ is the *i*th cardiac interval in ECG and $t_n$ denotes the occurrence of *n*th R wave.

### SCG signal processing

Heart beat detection on seismocardiograms is based on nearly periodic appearance of fiducial points in SCG signal [35]. We chose Aortic valve opening (AO) wave which indicates the start of ventricular contraction and is usually visible as a single sharp wave [19].

In this study, we compare two beat detection algorithms: beat detection algorithm proposed by Tadi et al. in paper [25] used as a reference method of heart beat detection and the heart beat detector on SCG signals described in paper [24] described further as the tested beat detector.

#### Reference beat detection algorithm

Algorithm presented by Tadi et al. in 2015 [25] uses R waves as reference points and is based on the windowing method proposed in papers [36, 37]. The first step of the algorithm is applying a band-pass filter with cut-off frequencies of 4 Hz and 50 Hz. Then, the SCG signal is smoothed using a moving average filter, whose window has the duration of 10–20 ms. The R waves in the ECG signal are localized using Pan–Tompkins algorithm and are the reference points. The location of AO wave of a cardiac cycle is determined as a maximum value of the SCG signal within a 90 ms window.

#### Tested heart beat detector

Beat detector proposed by Tadi et al. in 2016 [24] consists of the following steps: applying band-pass filtering to the signal (3rd order Butterworth filter with cut-off frequencies of 1 Hz and 45 Hz), motion noise cancellation, Hilbert transform and applying band-pass filter with cut-off frequencies of 0.5 Hz and 3 Hz to obtain a waveform with the same periodicity as heart rate.

Motion noise detection consists of calculating signal power envelope, and thresholding. Signal power envelope is calculated from the SCG signal using root mean square operation and a sliding window with a length of 500 ms. Signal parts, where the power envelope exceeds the threshold (twice the median value of signal power envelope), are classified as motion artifacts.

According to Tadi et al. [24], Hilbert transform improves the heart beat detection in SCG signals, because it facilitates the detection of the dominant peaks associated with heart beats. The envelope of the signal *s*(*t*) can be obtained by applying the Hilbert transform defined in the following equation:

2 $$\begin{aligned} \hat{s}(t) = \frac{1}{\pi } \int _{-\infty }^{+\infty } \frac{s(\tau )}{t - \tau } \mathrm {d}\tau . \end{aligned}$$

Hilbert transform yields a $90^{\circ }$ phase shift of *s*(*t*) and thus we can calculate the magnitude of its envelope as in the following equation:

3 $$\begin{aligned} A(t) = |s_a (t)| = \sqrt{s^2 (t) + \hat{s}^2 (t)}, \end{aligned}$$

where $s_a(t)$ is an analytic signal.

In the last step, we find local maxima of the magnitude of Hilbert envelope separated by at least 400 ms. These maxima determine the positions of AO waves. The interbeat intervals in SCG are calculated as differences between timing points of successive AO waves as in the following equation:

4 $$\begin{aligned} t_{{\text{AO}-\text{AO}}, i} = t_n - t_{n-1}, \end{aligned}$$

where $t_{\text{AO},i}$ is the *i*th cardiac interval in SCG and $t_n$ denotes the occurrence of *n*th AO wave.

### HRV analysis

We calculated the mean interbeat interval (mean NN), the standard deviation of all interbeat intervals (SDNN), the ratio of number of interbeat interval differences greater than 50 ms (NN50), the proportion calculated by dividing NN50 (pNN50) by the total number of interbeat intervals, the root mean square of differences (RMSSD) of successive RR intervals in accordance with current recommendations [2]. For frequency domain analysis, we used sampling frequency equal to 3 Hz and Hann window defined in the following equation:

5 $$\begin{aligned} w(n) = \frac{1}{2} \left( 1 - \cos \left( 2 \pi \frac{n}{N} \right) \right) , \end{aligned}$$

where $N = L - 1$, *L* is the window length, and $0 \le n \le N$ [38].

The power of the low-frequency band (${{\text{PSD}}}_{{\text{LF}}}$) was computed in the band 0.04–0.15 Hz, the power of very low-frequency band (${{\text{PSD}}}_{{\text{VLF}}}$) was calculated for frequencies under 0.04 Hz, and the power of the high frequency band (${{\text{PSD}}}_{{\text{HF}}}$) was computed in the band 0.15–0.4 Hz. The LF/HF ratio was computed as the ${{\text{PSD}}}_{{\text{LF}}}$/${{\text{PSD}}}_{{\text{HF}}}$ ratio.

## Results

Due to the lack of annotations of recordings from CEBS database [39], the heart beats in SCG signal were annotated using the algorithm described in “Reference beat detection algorithm”. Heart beats determined by this algorithm are treated as reference beats for SCG signal. Tested heart beat detector based on algorithm proposed in paper [24] was evaluated as the number of true positives (TP), false positives (FP), false negatives (FN), the number of beats, sensitivity, and positive predictive value (precision).

When the difference between position of reference AO wave and detected AO wave is within 180 ms margin, then this AO wave position is considered a true positive. False negative occurs when tested beat detector omits a true AO wave in reference annotation. False positive is determined for false detected AO wave.

Sensitivity (Se) is defined in the following equation:

6 $$\begin{aligned} \text{Se} = \frac{{\text{TP}}}{{\text{TP+FN}}}, \end{aligned}$$

and positive predictive value (PPV) is defined in the following equation:

7 $$\begin{aligned} \text{PPV} = \frac{{\text{TP}}}{{\text{TP+FP}}}. \end{aligned}$$

The number of beats is the sum of TP and FN. Table 1 presents beat detector performance measures on signals b001–b020. Table 2 presents beat detector performance measures on signals m001–m020 and Table 3 shows performance measures on signals p001–p020.

**Table 1:** Performance measures of tested heart beat detector on SCG signals b001–b020

Signal	TP	FP	FN	Beats	Se	PPV
b001	279	22	19	298	0.936	0.927
b002	308	0	0	308	1.000	1.000
b003	121	187	226	347	0.351	0.395
b004	323	2	1	325	0.997	0.994
b005	139	226	224	364	0.385	0.383
b006	309	2	0	309	1.000	0.994
b007	272	1	0	272	1.000	0.996
b008	480	0	1	480	0.998	1.000
b009	310	6	3	313	0.990	0.981
b010	234	75	74	309	0.761	0.758
b011	251	86	86	338	0.746	0.741
b012	317	81	86	403	0.787	0.797
b013	358	0	1	359	0.997	1.000
b014	345	1	0	345	1.000	0.997
b015	329	3	1	330	0.997	0.991
b016	352	0	0	352	1.000	1.000
b017	363	2	2	365	0.995	0.995
b018	400	0	0	400	1.000	1.000
b019	316	0	0	338	1.000	1.000
b020	338	15	12	338	0.965	0.956
Total	6137	711	736	6873	0.893	0.896

**Table 2:** Performance measures of tested heart beat detector on SCG signals m001–m020

Signal	TP	FP	FN	Beats	Se	PPV
m001	3794	129	113	3907	0.971	0.967
m002	3205	20	20	3225	0.994	0.994
m003	2283	339	836	3119	0.732	0.871
m004	3404	22	3	3407	0.999	0.994
m005	1949	1656	1650	3599	0.542	0.541
m006	3086	32	30	3116	0.990	0.990
m007	2596	23	18	2614	0.993	0.991
m008	5008	3	11	5019	0.998	0.999
m009	2949	234	211	3160	0.933	0.926
m010	2148	842	840	2988	0.719	0.718
m011	3450	148	146	3596	0.959	0.959
m012	3744	233	246	3990	0.938	0.941
m013	3707	5	4	3711	0.999	0.999
m014	3378	39	39	3417	0.989	0.989
m015	3204	2	1	3205	1.000	0.999
m016	3860	2	1	3861	1.000	0.999
m017	3574	11	12	3586	0.997	0.997
m018	4011	69	93	4104	0.977	0.983
m019	3178	14	17	3195	0.995	0.996
m020	3386	15	12	3398	0.996	0.996
Total	65,914	3838	4303	70,217	0.939	0.945

**Table 3:** Performance measures of tested heart beat detector on SCG signals p001–p020

Signal	TP	FP	FN	Beats	Se	PPV
p001	317	11	8	325	0.975	0.966
p002	308	0	0	308	1.000	1.000
p003	95	254	253	348	0.273	0.272
p004	324	2	1	325	0.997	0.994
p005	181	240	184	365	0.496	0.430
p006	139	226	224	272	0.176	0.381
p007	273	1	0	273	1.000	0.996
p008	479	0	1	480	0.998	1.000
p009	313	6	3	316	0.991	0.981
p010	234	75	74	308	0.760	0.757
p011	251	88	86	337	0.745	0.740
p012	317	81	87	404	0.785	0.796
p013	358	0	1	359	0.997	1.000
p014	344	1	0	344	1.000	0.997
p015	328	3	1	329	0.997	0.991
p016	352	0	0	352	1.000	1.000
p017	363	2	2	365	0.995	0.995
p018	400	0	0	400	1.000	1.000
p019	316	0	0	316	1.000	1.000
p020	326	15	12	338	0.964	0.956
Total	6018	1005	937	6864	0.877	0.857

The best heart beat detection performance of tested algorithm within the analyzed series was achieved on signals m001–m020 (overall sensitivity of 0.939 and positive predictive value of 0.945) due to the lower number of false positive and false negative results. The worst overall performance was achieved on signals p001–p020 (overall sensitivity of 0.877, precision of 0.857) and the performance of heart beat detection expressed as the overall sensitivity was 0.893 and for overall precision value of 0.896.

Among the individual signals, the best results were achieved for recordings b002, b018, b019, p002, p016, p018, and p019 ($\text {Se}=1.000$, $\text {PPV}=1.000$). The worst results were obtained for signal p006 ($\text {Se}=0.176$, $\text {PPV}=0.381$), p003 ($\text {Se}=0.273$, $\text {PPV}=0.272$), b003 ($\text {Se}=0.351$, $\text {PPV}=0.395$), and b005 ($\text {Se}=0.385$, $\text {PPV}=0.383$) because of high levels of FP and FN which were caused by motion artifacts and the fact that the AO wave was not always the most prominent peak of the signal.

Mean and standard deviations of HRV indices obtained from interbeat intervals from ECG and SCG are presented in Table 4 for signals b001–b020, for signals m001–m020 in Table 5, in Table 6 for signals p001–p020, and in Table 7 for all analyzed signals.

**Table 4:** HRV indices derived from ECG lead I and SCG signal presented as mean and standard deviation (SD) on recordings b001–b020

HRV index	ECG		SCG (reference algorithm)		SCG (tested algorithm)	
	Mean	SD	Mean	SD	Mean	SD
Mean NN [ms]	880.6236	102.1249	880.6352	102.1375	877.9416	100.0651
SDNN [ms]	55.3286	18.0816	58.8625	16.7121	92.5927	55.8870
RMSSD [ms]	18.4905	21.1768	59.2054	20.4972	116.8905	100.4839
NN50	74.8000	46.9093	107.8000	42.1059	138.2500	84.8211
pNN50	0.2240	0.1408	0.3233	0.1319	0.4099	0.2501
${{\text{PSD}}}_{{\text{LF}}}$ [$\text {ms}^{2}$]	616,229.8079	155,950.1659	612,073.3519	155,948.3816	612,073.3519	154,059.9771
${{\text{PSD}}}_{{\text{VLF}}}$ [ms2]	2962.8256	2308.0362	3301.8585	2148.7678	10,018.8269	15,211.3318
${{\text{PSD}}}_{{\text{HF}}}$ [$\text {ms}^{2}$]	616,714.3314	155,958.1736	616,713.8099	155,953.0616	612,867.8984	153,989.0784
LF/HF	0.9992	0.0006	0.9992	0.0006	0.9986	0.0022

**Table 5:** HRV indices derived from ECG lead I and SCG signal presented as mean and standard deviation (SD) on recordings m001–m020

HRV index	ECG		SCG (reference algorithm)		SCG (tested algorithm)	
	Mean	SD	Mean	SD	Mean	SD
Mean NN [ms]	868.4482	107.7381	868.4494	107.7377	874.4701	108.1814
SDNN [ms]	66.7848	27.6628	69.4776	27.2620	146.1656	259.5451
RMSSD [ms]	54.2829	33.0621	63.9567	31.9611	177.9677	335.4112
NN50	699.5000	375.9997	1051.6500	480.1267	1223.8000	689.7342
pNN50	0.2068	0.1085	0.3098	0.1433	0.3621	0.2137
${{\text{PSD}}}_{{\text{LF}}}$ [$\text {ms}^{2}$]	564,775.7879	150,123.2398	564,774.1763	150,124.8666	560,361.6889	153,743.7541
${{\text{PSD}}}_{{\text{VLF}}}$ [$\text {ms}^{2}$]	2730.3091	1914.1473	3076.3276	150,124.8666	7566.0048	8040.7530
${{\text{PSD}}}_{{\text{HF}}}$ [$\text {ms}^{2}$]	565,323.1825	150,225.7814	565,321.7553	150,228.3664	561,047.5560	153,853.2790
LF/HF	0.9990	0.0005	0.9990	0.0005	0.9987	0.0008

**Table 6:** HRV indices derived from ECG lead I and SCG signal presented as mean and standard deviation (SD) on recordings p001–p020

HRV index	ECG		SCG (reference algorithm)		SCG (tested algorithm)	
	Mean	SD	Mean	SD	Mean	SD
Mean NN [ms]	881.2197	102.3434	881.2311	102.3560	879.9381	100.3830
SDNN [ms]	55.6498	17.7449	58.9799	16.5574	84.3181	34.9819
RMSSD [ms]	48.4851	21.1828	58.4208	21.0497	103.2012	69.7998
NN50	168.1500	23.8311	167.8500	22.4365	132.8000	81.0884
pNN50	0.4899	0.0414	0.4892	0.0347	0.3911	0.2336
${{\text{PSD}}}_{{\text{LF}}}$ [$\text {ms}^{2}$]	616,967.1499	156,145.0747	616,968.3450	156,144.2675	616,229.7384	153,878.4562
${{\text{PSD}}}_{{\text{VLF}}}$ [$\text {ms}^{2}$]	2974.3376	2298.2310	3300.7693	2145.1549	7458.9407	7783.3257
${{\text{PSD}}}_{{\text{HF}}}$ [$\text {ms}^{2}$]	617,450.7037	156,152.3237	617,453.1540	156,148.1566	616,747.0991	153,885.5876
LF/HF	0.9992	0.0006	0.9992	0.0006	0.9991	0.0006

**Table 7:** HRV indices derived from ECG lead I and SCG signal presented as mean and standard deviation (SD) on all analyzed recordings

HRV index	ECG		SCG (reference algorithm)		SCG (tested algorithm)	
	Mean	SD	Mean	SD	Mean	SD
Mean NN [ms]	876.7638	102.4935	876.7719	102.5018	874.4701	108.1814
SDNN [ms]	59.2544	21.9539	62.4400	21.0417	146.1656	259.5451
RMSSD [ms]	50.4195	25.4661	62.4400	24.7629	177.9677	335.4112
NN50	688.3000	754.5296	686.0833	750.9212	1223.800	689.7342
pNN50	0.4919	0.0366	0.4924	0.0353	0.3621	0.2137
${{\text{PSD}}}_{{\text{LF}}}$ [$\text {ms}^{2}$]	599,324.2486	153,454.5209	607,119.5110	161,038.1290	560,361.6889	153,743.7541
${{\text{PSD}}}_{{\text{VLF}}}$ [$\text {ms}^{2}$]	2889.1574	2146.9039	3226.3185	2003.5468	7566.0048	8040.7530
${{\text{PSD}}}_{{\text{HF}}}$ [$\text {ms}^{2}$]	599,829.4059	153,486.9967	607,626.5532	161,052.9574	561,047.5560	153,853.2790
LF/HF	0.9991	0.0006	0.9991	0.0006	0.9987	0.0008

Mean and standard deviation values of calculated indices are similar in each group of signals except SDNN, RMSSD, NN50, pNN50, and ${{\text{PSD}}}_{{\text{VLF}}}$, where values achieved for tested algorithm are significantly greater. HRV indices mean and standard deviation are similar for 5-min signals (b001–b020 and p001–p020).

Tadi et al. [25] observed that HRV indices obtained from ECG and SCG have strong linear relationship. To examine the strength of linear correlation between HRV indices obtained from ECG and SCG, we used MATLAB Curve Fitting Tool to calculate the goodness of fit to the 1st degree polynomial (linear) model. The goodness of fit is expressed as the coefficient of determination $R^2$.

**Fig. 1:**
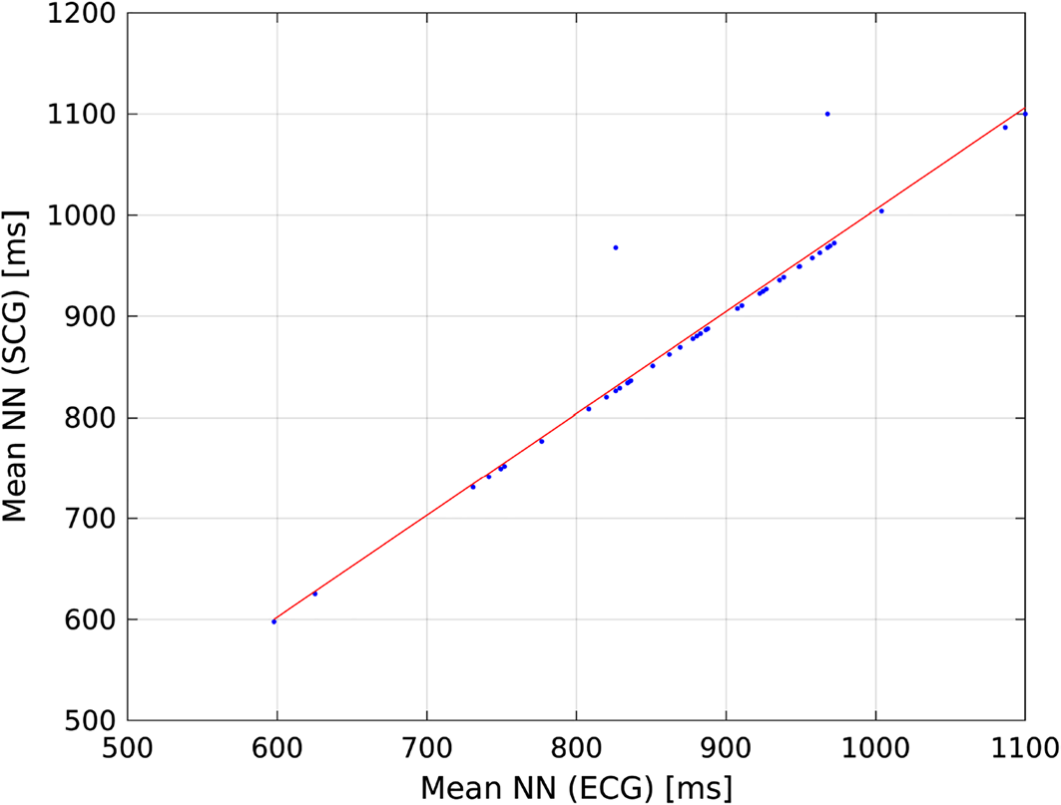
Linear model describing the correlation between mean NN derived from SCG and mean NN calculated from SCG using reference algorithm

**Fig. 2:**
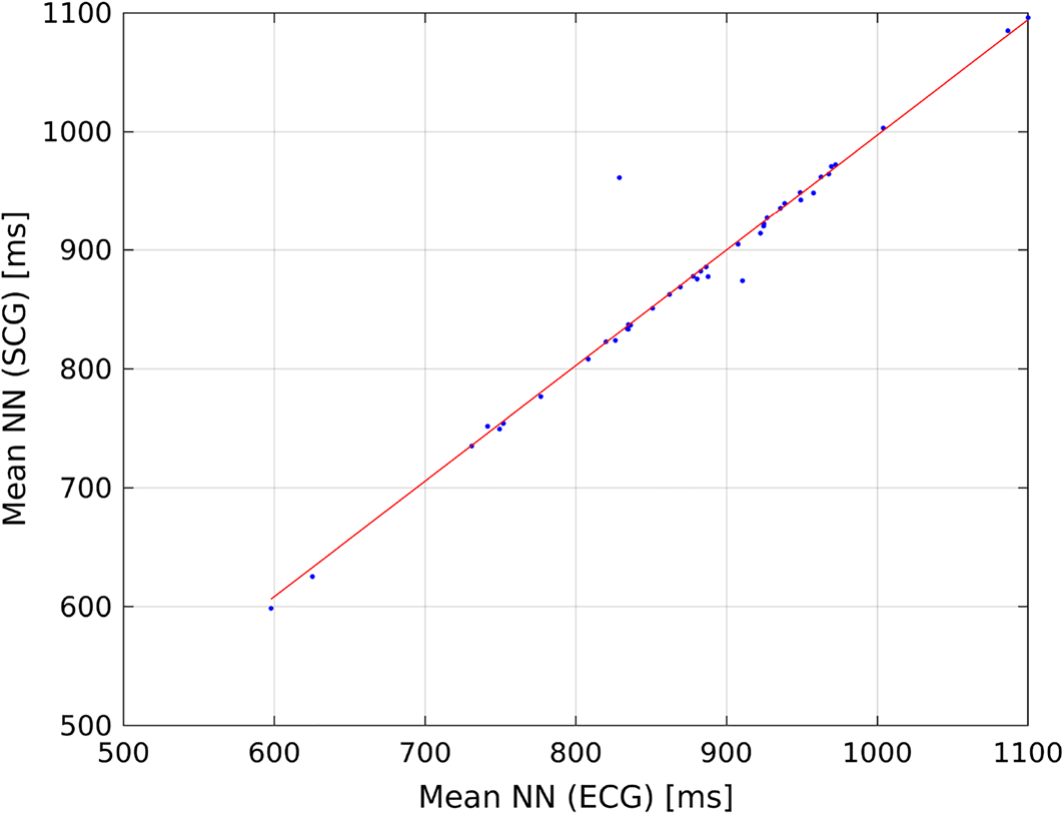
Linear model describing the correlation between mean NN derived from SCG and mean NN calculated from SCG using tested algorithm

**Fig. 3:**
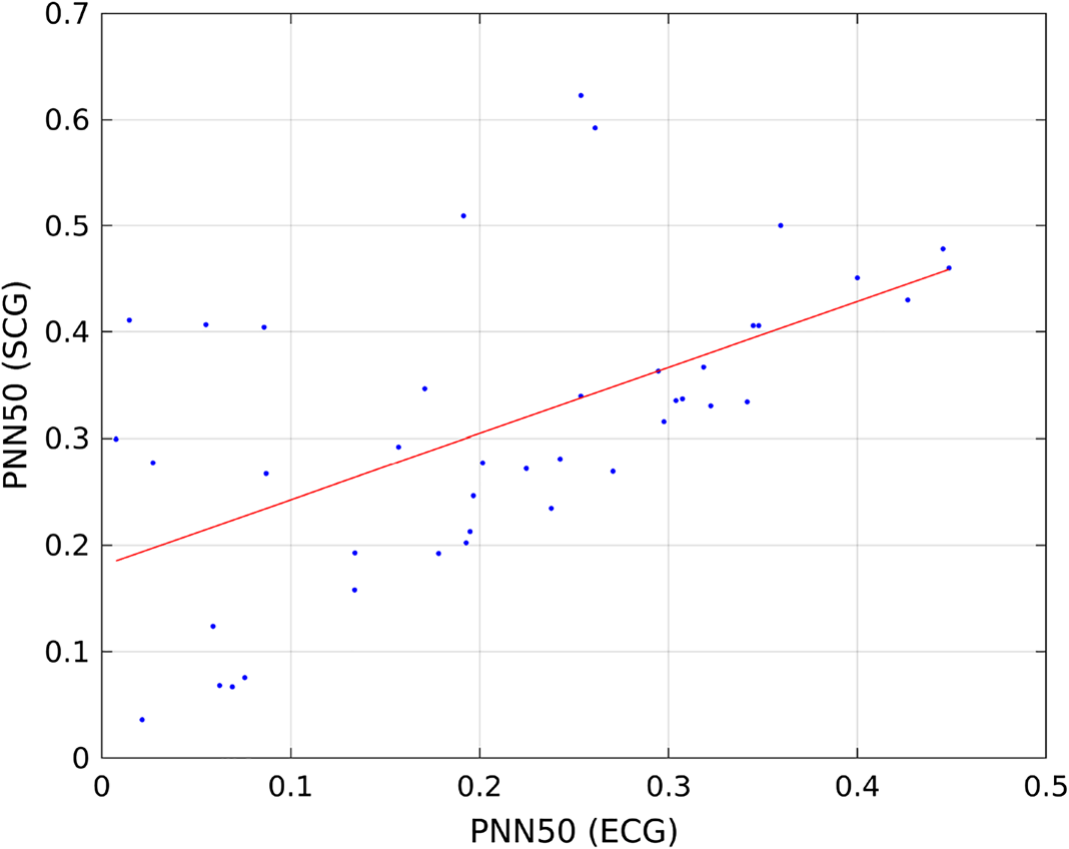
Linear model describing the correlation between pNN50 derived from SCG and pNN50 calculated from SCG using reference algorithm

**Fig. 4:**
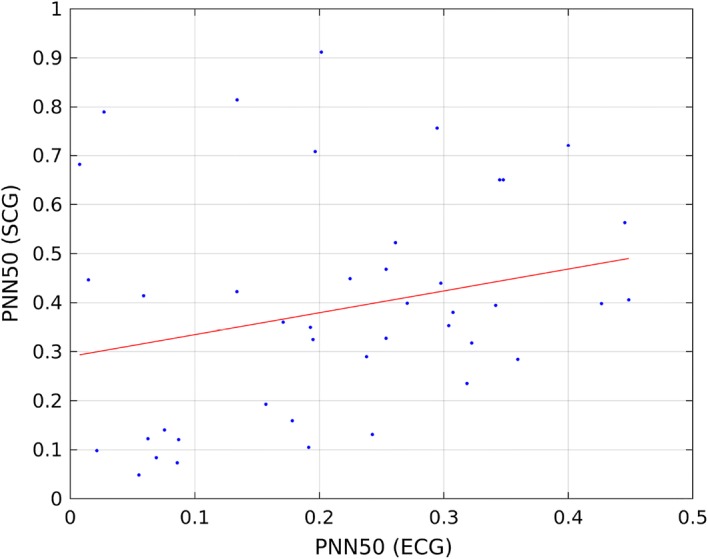
Linear model describing the correlation between pNN50 derived from SCG and pNN50 calculated from SCG using tested algorithm

Table 8 presents, Tables 9, 10, and 11 present correlation of determination ($R^2$) calculated for linear model describing the relationship of HRV indices calculated from ECG and SCG on recordings b001–b020, m001–m020, p001–p020, and all recordings. Figures 1 and 2 present linear model describing the relationship between mean NN calculated from ECG and SCG, and Figs. 3 and 4 present linear model of pNN50 derived from ECG and SCG.

**Table 8:** Correlation between HRV indices obtained from ECG and SCG on recordings b001–b020

HRV index	$R^2$ (reference algorithm)	$R^2$ (tested algorithm)
Mean NN	1.0000	0.9925
SDNN	0.9263	0.0533
RMSSD	0.5983	0.0546
NN50	0.3566	0.0133
pNN50	0.3854	0.0168
${{\text{PSD}}}_{{\text{LF}}}$	1.0000	0.9844
${{\text{PSD}}}_{{\text{VLF}}}$	0.9379	0.0416
${{\text{PSD}}}_{{\text{HF}}}$	1.0000	0.9862
LF/HF	0.9977	0.0360

**Table 9:** Correlation between HRV indices obtained from ECG and SCG on recordings m001–m020

HRV index	$R^2$ (reference algorithm)	$R^2$ (tested algorithm)
Mean NN	1.0000	0.9249
SDNN	0.9263	0.5507
RMSSD	0.5983	0.4898
NN50	0.1791	0.1166
pNN50	0.2137	0.1580
${{\text{PSD}}}_{{\text{LF}}}$	1.0000	0.9846
${{\text{PSD}}}_{{\text{VLF}}}$	0.8967	0.0009
${{\text{PSD}}}_{{\text{HF}}}$	1.0000	0.9846
LF/HF	0.9996	0.4296

**Table 10:** Correlation between HRV indices obtained from ECG and SCG on recordings p001–p020

HRV index	$R^2$ (reference algorithm)	$R^2$ (tested algorithm)
Mean NN	1.0000	0.9984
SDNN	0.9232	0.0043
RMSSD	0.9536	0.0176
NN50	0.8957	0.0504
pNN50	0.6782	0.0684
${{\text{PSD}}}_{{\text{LF}}}$	1.0000	0.9980
${{\text{PSD}}}_{{\text{VLF}}}$	1.0000	0.0094
${{\text{PSD}}}_{{\text{HF}}}$	1.0000	0.9980
LF/HF	0.9976	0.9629

**Table 11:** Correlation between HRV indices obtained from ECG and SCG on all recordings

HRV index	$R^2$ (reference algorithm)	$R^2$ (tested algorithm)
Mean NN	1.0000	0.9681
SDNN	0.9232	0.2738
RMSSD	0.6092	0.2047
NN50	0.3949	0.5800
pNN50	0.4410	0.0617
${{\text{PSD}}}_{{\text{LF}}}$	1.0000	0.9889
${{\text{PSD}}}_{{\text{VLF}}}$	0.9390	0.0132
${{\text{PSD}}}_{{\text{HF}}}$	1.0000	0.9895
LF/HF	0.9976	0.1326

$R^2$ values calculated for linear fit between HRV indices derived from ECG and reference SCG beats indicate strong linear relationship except for NN50 and pNN50 in all signal groups except signals p001–p020 for NN50. When using tested heart beat detector, obtained $R^2$ values are lower for all recording groups. Mean NN, ${{\text{PSD}}}_{{\text{LF}}}$ and ${{\text{PSD}}}_{{\text{HF}}}$ have the maximum value of $R^2$ for each group of recordings. The weakest correlation was observed for SDNN, RMSSD, PNN50, and LF/HF for all groups of recordings, except LF/HF for recordings p001–p020.

Despite the similarities of mean and standard deviation of analyzed HRV indices among the analyzed signals (heart beats obtained from ECG lead I, reference SCG beats and SCG with heart beats obtained using tested algorithm), there are significant differences in correlation between HRV indices between ECG and SCG signals. These discrepancies show that the correlation between HRV indices obtained from ECG and SCG depends on the quality of heart beat detection on SCG signals. To reduce the influence of the outliers in the model which are shown in Figs. 2 and 4, we applied robust model fitting using least absolute residual (LAR) method described further in [40] and least squares method (LSq). Then, the maximum value of $R^2$ coefficient was chosen as a result of robust model fitting. $R^2$ coefficients for recordings b001–b020 are shown in Table 12, for recordings m001–m020 are presented in Table 13 and $R^2$ values for recordings p001–p020 are shown in Table 14. Table 15 presents the $R^2$ values for all analyzed recordings.

**Table 12:** Correlation between HRV indices obtained from ECG and SCG on recordings b001–b020 using robust linear model

HRV index	$R^2$ value	Robust model fit method
Mean NN	0.9987	LAR
SDNN	0.8422	LAR
RMSSD	0.8424	LSq
NN50	0.8356	LAR
pNN50	0.8271	LAR
${{\text{PSD}}}_{{\text{LF}}}$	0.9977	LSq
${{\text{PSD}}}_{{\text{VLF}}}$	0.8403	LAR
${{\text{PSD}}}_{{\text{HF}}}$	0.9977	LSq
LF/HF	0.8393	LAR

**Table 13:** Correlation between HRV indices obtained from ECG and SCG on recordings m001–m020 using robust linear model

HRV index	$R^2$ value	Robust model fit method
Mean NN	0.9875	LAR
SDNN	0.9251	LAR
RMSSD	0.8424	LSq
NN50	0.2114	LSq
pNN50	0.3109	LSq
${{\text{PSD}}}_{{\text{LF}}}$	0.9974	LSq
${{\text{PSD}}}_{{\text{VLF}}}$	0.8355	LAR
${{\text{PSD}}}_{{\text{HF}}}$	0.9974	LSq
LF/HF	0.8997	LAR

**Table 14:** Correlation between HRV indices obtained from ECG and SCG on recordings p001–p020 using robust linear model

HRV index	$R^2$ value	Robust model fit method
Mean NN	0.9984	LSq
SDNN	0.8354	LAR
RMSSD	0.1436	LSq
NN50	0.8417	LAR
pNN50	− 0.0893	LAR
${{\text{PSD}}}_{{\text{LF}}}$	0.9997	LSq
${{\text{PSD}}}_{{\text{VLF}}}$	0.8349	LAR
${{\text{PSD}}}_{{\text{HF}}}$	0.9997	LSq
LF/HF	0.9938	LAR

**Table 15:** Correlation between HRV indices obtained from ECG and SCG on all recordings using robust linear model

HRV index	$R^2$ value	Robust model fit method
Mean NN	0.9970	LAR
SDNN	0.9398	LAR
RMSSD	0.9377	LSq
NN50	0.9374	LAR
pNN50	− 0.0199	LAR
${{\text{PSD}}}_{{\text{LF}}}$	0.9951	LAR
${{\text{PSD}}}_{{\text{VLF}}}$	0.9390	LAR
${{\text{PSD}}}_{{\text{HF}}}$	0.9950	LAR
LF/HF	0.9432	LAR

Using robust model fitting improves the correlation between HRV indices obtained from ECG and SCG except for* p* series and all analyzed signals due to the large number of outliers found in linear model.

## Conclusion

In this study, we presented the feasibility of HRV analysis using SCG signal and compared results obtained from ECG and SCG heart beats. Mean interbeat interval (Mean NN), ${{\text{PSD}}}_{{\text{LF}}}$ and ${{\text{PSD}}}_{{\text{HF}}}$ are most robust to SCG signal noise and have the strongest linear correlation. HRV indices obtained from heart beat intervals using two different beat detectors on SCG signals are similar except SDNN, RMSSD, NN50, pNN50, and ${{\text{PSD}}}_{{\text{VLF}}}$, which are induced by noise in SCG signals and the limitations of tested beat detector. Using robust fitting to the linear model improves the correlation between HRV indices obtained from ECG and SCG signals [28] (except the $R^2$ values of pNN50 values in* p* series and for all analyzed signals) and indicates the need to design a reliable heart beat detector which works on SCG signals.

Beat detection performance of tested algorithm on all SCG signals is quite good on 85,954 beats ($\text {Se}=0.930$, $\text {PPV}=0.934$) despite lower performance on noisy signals. Sensitivity and PPV on signals b001–b020 ($\text {Se}=0.893$, $\text {PPV}=0.896$) is lower than reported in paper [28] ($\text {Se}=0.995$, $\text {PPV}=0.991$) and Rivero et al. paper [41] ($\text {Se}=0.99$, $\text {PPV}=0.97$). Tested algorithm has lower performance on SCG signals m001–m020 than reported in Li et al. [39] paper ($\text {Se}=0.9933$, $\text {PPV}=0.9941$) due to the fact that tested algorithm based on the beat detector proposed by Tadi et al. [24] was susceptible to noise which strongly worsens the performance of heart beat detection. Other causes of worse performance include the occurrence of AO waves which are not the most prominent peaks and the fact that the heart beats on SCG signals detected as the nearest local maximum after the occurrence of the R wave in ECG signal may not occur within 90 ms.

Strong linear relationship between most HRV indices obtained from ECG and SCG signals, especially between indices derived from ECG and reference beat detector on SCG signals, indicates the reliability of using SCG-derived interbeat intervals for HRV analysis [18, 25, 28]. Lower coefficients of determination between HRV indices obtained from ECG signal and beats detected on SCG signal using tested algorithm are caused by the noise found in analyzed signals. The possibility of recording and processing cardiac vibrations using one device broadens the scope of applicability of SCG [18, 25]. HRV analysis on SCG signal performed on smartphones may be used in mental stress assessment [27] or atrial fibrillation detection [42]. In future works, we will investigate the influence of other SCG beat detection algorithms on HRV indices.

## Data Availability

The datasets used and/or analyzed during the current study are available from the corresponding author on reasonable request.

## Abbreviations

SCG: seismocardiography, seismocardiogram
HRV: heart rate variability
ECG: electrocardiogram
CEBS: combined measurement of ECG, breathing and seismocardiogram
AO: atrial valve opening wave
mean NN: mean interbeat interval
SDNN: standard deviation of all interbeat intervals
NN50: the ratio of number of interbeat intervals greater than 50 ms
pNN50: the ratio defined as the division of NN50 by the total number of interbeat intervals
RMSSD: root mean square of successful differences of interbeat intervals
${{\text{PSD}}}_{{\text{LF}}}$: the power of low-frequency band of interbeat intervals
${{\text{PSD}}}_{{\text{VLF}}}$: the power of very low-frequency band of interbeat intervals
${{\text{PSD}}}_{{\text{HF}}}$: the power of high frequency band of interbeat intervals
LF/HF: the ratio of ${{\text{PSD}}}_{{\text{LF}}}$ and ${{\text{PSD}}}_{{\text{HF}}}$
TP: true positive
FN: false negative
FP: false positive
Se: sensitivity
PPV: positive predictive value
$R^2$: coefficient of determination of linear regression fit
LAR: least absolute residual method
LSq: least squares method
